# Blood flow velocity in monocular retinoblastoma assessed by color doppler

**DOI:** 10.6061/clinics/2015(12)06

**Published:** 2015-12

**Authors:** Maria Teresa B C Bonanomi, Osmar C Saito, Patricia Picciarelli de Lima, Roberta Chizzotti Bonanomi, Maria Cristina Chammas

**Affiliations:** I Hospital das Clínicas da Faculdade de Medicina da Universidade de São Paulo, Departamento de Oftalmologia, São Paulo/SP, Brazil.; II Hospital das Clínicas da Faculdade de Medicina da Universidade de São Paulo, Departamento de Radiologia e Ultrassom, São Paulo/SP, Brazil.; III Hospital das Clínicas da Faculdade de Medicina da Universidade de São Paulo, Departamento de Patologia, São Paulo/SP, Brazil.; IV São Francisco Medical School, Oftalmologia, Bragança Paulista/SP, Brazil.

**Keywords:** Retinoblastoma, Color Doppler Ultrasonography, Blood Flow Velocity, Eye Enucleation, Histopathology

## Abstract

**OBJECTIVE:**

To analyze the flow of retrobulbar vessels in retinoblastoma by color Doppler imaging.

**METHODS:**

A prospective study of monocular retinoblastoma treated by enucleation between 2010 and 2014. The examination comprised fundoscopy, magnetic resonance imaging, ultrasonography and color Doppler imaging. The peak blood velocities in the central retinal artery and central retinal vein of tumor-containing eyes (tuCRAv and tuCRVv, respectively) were assessed. The velocities were compared with those for normal eyes (nlCRAv and nlCRVv) and correlated with clinical and pathological findings. Tumor dimensions in the pathological sections were compared with those in magnetic resonance imaging and ultrasonography and were correlated with tuCRAv and tuCRVv. In tumor-containing eyes, the resistivity index in the central retinal artery and the pulse index in the central retinal vein were studied in relation to all variables.

**RESULTS:**

Eighteen patients were included. Comparisons between tuCRAv and nlCRAv and between tuCRVv and nlCRVv revealed higher velocities in tumor-containing eyes (*p*<0.001 for both), with a greater effect in the central retinal artery than in the central retinal vein (*p*=0.024). Magnetic resonance imaging and ultrasonography measurements were as reliable as pathology assessments (*p*=0.675 and *p*=0.375, respectively). A positive relationship was found between tuCRAv and the tumor volume (*p*=0.027). The pulse index in the central retinal vein was lower in male patients (*p*=0.017) and in eyes with optic nerve invasion (*p*=0.0088).

**CONCLUSIONS:**

TuCRAv and tuCRVv are higher in tumor-containing eyes than in normal eyes. Magnetic resonance imaging and ultrasonography measurements are reliable. The tumor volume is correlated with a higher tuCRAv and a reduced pulse in the central retinal vein is correlated with male sex and optic nerve invasion.

## INTRODUCTION

Retinoblastoma is a highly malignant ocular neoplasm that tends to progress to optic disc invasion 1, which suggests a poor prognosis for the patient 2. The diagnosis of nerve invasion at patient presentation is important for prognostication and management 3. Magnetic resonance imaging (MRI) is used to confirm the presence of a tumor inside the eye, to determine the extent of the tumor within the optic nerve and the brain as well as to detect associated primary intracranial pinealoma 4.

Although MRI is the gold standard for detecting nerve invasion and is recommended for every child suspected of harboring a retinoblastoma 5, it can be ineffective for this purpose. This problem is particularly an issue in non-enlarged optic nerves 6, despite the application of special techniques 7. A recent meta-analysis showed a sensitivity of only 53% for optic nerve invasion (ONi), indicating a large number of false-negative results 8.

Ocular ultrasonography (US) is a commonly used technique for confirming irregular retinoblastoma masses inside the eye. US can be performed without sedation and allows for the accurate visualization of calcium inside the tumor 9. Computed tomography is unnecessary for initial diagnostic assessments because when coupled with MRI, US is currently the safest and best method to diagnose retinoblastoma.

Color Doppler imaging (CDI) is a US technique that combines B-scan images with the velocity information obtained from the Doppler shift of moving erythrocytes at a known frequency; this method may be used to study the small vessels of the orbit 10. Both the blood velocity and the presence of vascular channels can be assessed, supplying data for calculating indices to better understand the flow patterns in retrobulbar blood vessels 11.

The purpose of this study was to use CDI to image retrobulbar blood vessels in monocular retinoblastoma before enucleation, to compare blood velocities in the central retinal artery (CRA) and central retinal vein (CRV) between a tumor-containing eye and the contralateral normal eye and to determine whether Doppler findings correlate with high-risk clinical and pathological features of the enucleated eye that could impact treatment and prognosis. A secondary objective was to compare MRI, US and pathology measurements of the tumor mass itself.

## METHODS

This prospective study examined all monocular retinoblastomas treated by enucleation without preoperative adjunctive therapy in patients who presented to the Hospital das Clínicas da Faculdade de Medicina da Universidade de São Paulo between August 2010 and January 2014. This study was approved by the institutional review board of the hospital. The indication for primary enucleation was based on the international classification of retinoblastoma 12,14, including only the advanced stages (D and E) of the disease, without a prognosis of vision recovery. The preoperative workup comprised orbital and cranial MRI, orbital US, CDI and ophthalmological examination with fundus drawing under sedation. The high-risk clinical signs studied were glaucoma, buphthalmos and proptosis.

**Figure 1:**
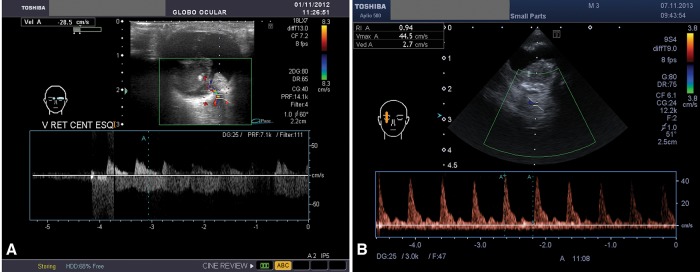
Color Doppler image coupled with a 16 MHz ultrasonography image of a unilateral retinoblastoma. The central retinal artery and central retinal vein were identified together in the middle of the optic nerve ultrasonography image and were then assessed from the posterior scleral surface (A) up to approximately 10 mm behind this landmark (B). The flow above the x-axis shows a peaked wave and represents the central retinal artery; the wave below this axis is more undulated and represents the central retinal vein. The spot assessed is denoted by the two parallel lines crossed by an oblique line to indicate the Doppler angle, which is ideally between 30° and 60°. The blue cross and blue ‘A’ were added manually to the wave to measure the velocity, which may be quite high in the central retinal artery of tumor-containing eyes (B).

**Figure 2:**
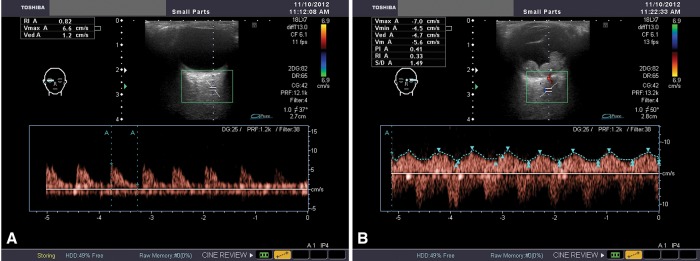
(A) Color Doppler image of the right eye, which was the contralateral normal eye, showing a reduced peak velocity in the central retinal artery and central retinal vein compared with that in tumor-containing eyes. (B) Color Doppler image of the left eye, which was the tumor-containing eye of the same patient as shown in (A); the velocities (min., max. and average), resistivity index and pulse index of the central retinal vein are displayed in the upper left-hand square. The wave boxes were inverted to allow automatic calculations for the vein. The blue ‘A’ and cross were added manually.

US and CDI were performed using a Toshiba Aplio XG and a Toshiba Aplio 500 (Tokyo, Japan) with a 16 MHz transducer with presets for small parts. The power output was 3-4 cm/sec and the mechanical index was set between 0.6 and 0.9; the power settings could not be reduced because doing so would have affected the velocity detection relative to the background noise artifact. The examination was performed along the longitudinal and transverse axes, with closed eyes and a large amount of US gel. Three tumor diameters were assessed three times to determine the arithmetic mean (longitudinal: L; transverse: T; and anterior-posterior: AP) and the volume was manually calculated based on these ultrasonographic means using the ellipsoid formula (L×T×AP×0.52). Blood flow was assessed in the CRA and CRV in both tumor-containing and normal eyes. The CRA and CRV were identified together in the middle of the optic nerve by using a US image and were measured from the posterior scleral surface up to approximately 10 mm behind this landmark using a Doppler angle between 30° and 60° (Figure 1). In a second approach that was only used for retinoblastoma-containing eyes, two indices were calculated and studied to evaluate high-risk clinical and pathological findings. The first was the Pourcelot resistive index, which is a measure of the peripheral vascular resistance of the CRA: RIa=(PSV-EDV)/PSV, where PSV is the peak systolic velocity and EDV is the end-diastolic velocity. The second was Gosling's pulsatility index for the CRV: PIv=(PSV-EDV)/MFV), where MFV is the mean of the PSV and EDV. The index calculation was performed automatically or manually after fixing the PSV and EDV (Figure 2). We chose RIa and PIv for the following reasons: the resistivity index for the CRA is highly reliable because higher velocities provide better reproducibility and the pulsatility index in the vein may provide an indication of nerve invasion because venous blood flow is influenced by the surrounding structures 11,15.

**Figure 3:**
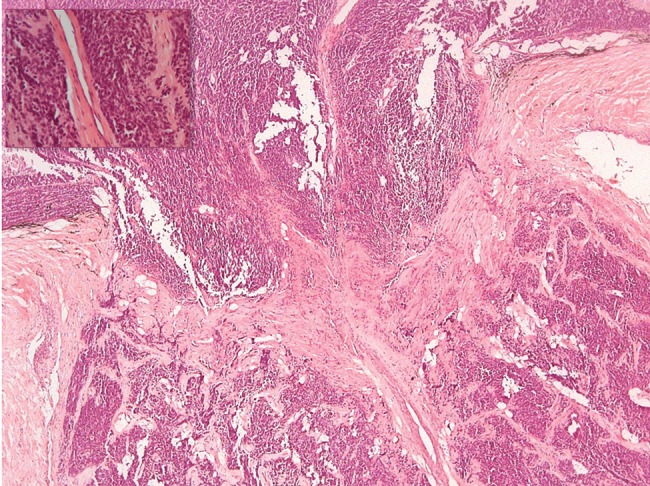
Pathology of an eye with enucleated retinoblastoma showing optic nerve invasion. The hematoxylin-eosin photomicrograph shows the posterior displacement of the lamina cribrosa by a compact blue tumor. Cords of tumor cells are intermingled with the retrobulbar nerve tissue. The central retinal vessels are surrounded by the invading tumor. Inset: higher-power magnification showing details of the central retinal vein surrounded by packed tumor cells.

The enucleated eye was immersed for 24 hours in 10% buffered neutral formalin. The surgical margin of the nerve was cut and embedded face-down in paraffin for sectioning. The globe was cut into three calottes, as follows: the main tumor block, lined antero-posteriorly with the pupil and the optic nerve; the temporal calotte; and the nasal calotte. The entire globe was then embedded in paraffin and a minimum of six serial sections (5 µm each) were cut at 100-150 µm intervals for each calotte. If necessary, additional sections were cut through the optic nerve head, the choroid and the optic nerve itself to assess the degree of invasion 16. The pieces were then processed for hematoxylin-eosin (HE) staining (Figure 3) and classified according to the pTNM American Joint Committee on Cancer (AJCC) classification 16. Retinoblastoma histopathological analysis was based on the College of American Pathologists (CAP) protocol 17, which involves examining the size of the tumor, the grade of differentiation and the degrees of necrosis and calcification. The antero-posterior axis plus both calottes were examined for risk features such as tumor invasion in the intraocular and extraocular tissues and optic nerve (ONi), prelaminar optic nerve (PreONi), postlaminar optic nerve (PosONi), surgical margin, anterior uveal, focal choroidal, massive (or larger than 3 mm) choroidal (mCHi), scleral and extrascleral invasion. Associated ocular findings secondary to the presence of the tumor, including goniosynechiae and iris neovascularization, were also analyzed.

The following experiments were performed. 1) The PSV in the CRA and the maximum velocity in the CRV were each compared between normal eyes (nlCRAv, nlCRVv) and tumor-containing eyes (tuCRAv, tuCRVv) and the arithmetic differences between the tumor-containing eye and the normal eye of a given patient were compared for the CRA and CRV. Clinical and pathological findings were also correlated with tuCRAv and tuCRVv. 2) The largest diameter in the pathology assessment and the largest diameters found using MRI and US were compared. 3) Correlations of tuCRAv and tuCRVv with the tumor volume (TUvol) determined by US and with MRI, US and pathology measurements were analyzed. 4) Correlations between PIv and RIa and all clinical and pathological features were analyzed. Statistical tests were conducted using SPSS 17.0 for Windows. The tests used (Student’s t-test, Pearson’s correlation, the nonparametric Mann-Whitney U test and linear regression) are specified for all of the results shown.

## RESULTS

In this period, we studied 18 cases that fulfilled the inclusion criteria; these cases are summarized in Table 1. Fourteen males (77.78%) and four females (22.22%) were included. The right eye (OD) was affected in eight cases (44.44%) and the left eye (OS) was affected in 10 (55.56%). Nine eyes were in the D group (50%) and nine were in the E group. Proptosis was present in two cases (11.11%) and glaucoma was present in seven cases (41.18%), four of which were complicated by buphthalmos (23.53%). Preoperative MRI showed a median MRIm of 18.35 mm (range, 10.6 to 28 mm). The images raised suspicion of ONi, as indicated by increased gadolinium uptake in four cases (22.22%), two CNS alterations, one pineal cyst and one probable intracanalicular ONi.

All CDI measurements were lost for one patient and the normal data (nlCRAv and nlCRVv) were lost for another three eyes due to a back-up failure in the machine. US measurements were recorded for all 18 eyes, with a median of 18.5 mm (range, 12.8-30 mm).

The pathology data showed a median diameter of 18.0 mm (range, 10-28 mm), with moderate differentiation in seven patients (38.89%), poor differentiation in 11 (61.11%) and demonstrable calcification in 16 (88.89%). Choroid invasion was demonstrated in 14 eyes (77.78%), but only six (33.33%) showed mCHi. Anterior uveal invasion was present in only one eye (5.56%). ONi was present in 11 eyes (61.11%), prelaminar invasion in 11 eyes (61.11%) and postlaminar invasion in four eyes (22.22%), with no compromised surgical margin. No extrascleral invasion was found, whereas scleral invasion was present in five eyes (27.78%). Iris neovascularization was present in 17 eyes (94.44%) and goniosynechiae was present in 15 eyes (83.33%).

Comparisons of the blood flow in normal eyes and retinoblastoma-containing eyes are shown in Table 2. The influence of tumor size and volume on the blood velocity is shown in Table 3. The only significant correlation was the positive correlation between tuCRAv and TUvol, as confirmed by the multivariate analysis (*p*=0.0331).

No difference was found in the tumor measurements by MRI or US compared with the pathology assessment (*p*=0.675 and *p*=0.375, respectively; Student’s t-test for matched pairs), indicating that both methods were able to reliably determine the real tumor size. However, the pathology assessment occasionally underestimated the real size due to fixative artifacts.

Age, sex, tumor stage (D or E), clinical complications (glaucoma, buphthalmos, proptosis), high-risk pathological features (ONi, surgical margin, anterior uveal, choroidal, mCHi, scleral and extrascleral invasion), tumor differentiation and necrosis and secondary pathological findings (iris neovascularization and goniosynechiae) did not modify the blood velocities in the CRA or CRV, as indicated by analysis using the parametric Mann-Whitney U test.

For only the tumor-containing eyes, RIa and PIv were also compared with all variables using the parametric Mann-Whitney U test. PIv was significantly lower in the male patients than in the female patients (*p*=0.0270). RIa and PIv were similar between eyes with clinical complications and those without them; among the pathological high-risk features, only ONi was correlated with a smaller PIv (*p*=0.0088), indicating that the venous pulse index was reduced when the optic nerve was invaded by the tumor (Table 4). The difference persisted when considering PreONi (*p*=0.0088) but not PosONi (*p*=0.2563). ONi did not modify the arterial index RIa (*p*=0.9596).

## DISCUSSION

Retinoblastoma is a highly malignant retinal neoplasia that tends to invade other intraocular structures and the optic nerve and, with progressive growth, to become extraocular. In recent years, due to educational programs, retinoblastoma has been diagnosed earlier, and retinoblastoma treatment has aimed to preserve the eye and the patient’s vision. Therefore, primary enucleation is indicated much less frequently than in the past and is only recommended for advanced stages because of the association with a higher risk of metastasis 14. For this reason, enucleation is the treatment of choice when there is little or no potential for vision recovery, especially if ONi is suspected. Important histopathological risk factors for local recurrence and metastasis include tumor invasion of the surgical margin of the optic nerve, postlaminar ONi, extrascleral invasion, scleral invasion and massive choroidal invasion 2,.

Imaging the eye prior to enucleation to predict tumor prognosis is a well-recognized technique. Imaging studies of the optic nerve, orbit and CNS using MRI may be important for both conservative treatment and enucleation of the eye 3; however, MRI findings in patients with normal-sized optic nerves have limited usefulness in preoperatively predicting the presence of ONi in retinoblastomas 6. According to a recent meta-analysis of radiological imaging of retinoblastomas that considered 591 eyes examined by MRI in 14 studies and found a sensitivity of 59% for postlaminar ONi, 74% for choroidal invasion and 88% for scleral invasion, MRI is an important diagnostic tool for determining the local tumor extent in advanced retinoblastoma. However, the diagnostic accuracy has room for improvement, especially regarding sensitivity 8.

Assessing the blood flow in intraocular tumors using CDI is not a new concept 21, but few prior publications are available. In a previous study, we analyzed vascularization inside a retinoblastoma itself by CDI as a follow-up to the conservative treatment of bilateral disease. Our findings showed that the blood vessels inside the tumor mass disappeared on the color Doppler image, indicating a response to the therapeutic approach, before total involution of the tumor. For this reason, this technique cannot replace fundus examination to evaluate the treatment of intraocular retinoblastoma but could be helpful for diagnosing, especially in the presence of cloudy ocular media and for evaluating the treatment response (unpublished data; Bonanomi MTBC, Saito OC, Tanaka T. Retinoblastoma assessment by Doppler sonography - a follow-up study. Poster presented at the Annual ARVO Meeting, May 2, 2011; Fort Lauderdale, Florida).

In the present study, we imaged retrobulbar vessels by CDI, compared retinoblastoma-containing eyes with the contralateral normal eyes and studied the relationships between blood velocities and demographics, clinical findings and tumor sizes and histopathological complications. We faced several difficulties in performing the examinations and analyzing the images. Even for CDI performed under sedation, obtaining at least three similar pulse waves that include calcium and its shadows for a tumor-containing eye can be challenging. Frequently, the limits of the sclera and optic nerve shadow are imprecise and the images must therefore be taken farther back in the optic nerve. Care must be taken to avoid placing the Doppler cursor more than 10 mm from the sclera because anatomically, the ophthalmic artery begins at 15 mm. If one overcomes this initial technical barrier, the measurements can be collected automatically. The peak blood velocities in the CRA and CRV were significantly higher in the tumor-containing eyes than in the contralateral normal eyes (Table 2), indicating a hemodynamic change in the retrobulbar vessels caused by the presence of a large tumor. The difference in the velocities between the normal eye and the tumor-containing eye of the same patient were larger in the CRA than in the CRV (Table 2), demonstrating that the tumor has a greater influence on tuCRAv than on tuCRVv.

In retinoblastoma-containing eyes, the next challenge was to calculate the volume in a diffuse infiltrative tumor or in multiple non-confluent tumors. In both cases, the sum of the volumes, calculated segment by segment, was considered as TUvol. This point is important because TUvol was the only size variable related to a higher velocity in the CRA (Table 3). Thus, according to this result, tumor-containing eyes presented higher peak velocities in the CRA and CRV. The difference was higher for the artery and the increase in tuCRAv was positively correlated with TUvol.

However, when tumor-containing eyes were considered exclusively, tuCRAv and tuCRVv were not influenced by age, sex, tumor stage, clinical complications (e.g., buphthalmos and proptosis) or any high-risk pathological features. Thus, according to these findings, the presence of a tumor alone, especially a large tumor, can trigger the sequence of events that causes elevations in the blood flow velocities in the retrobulbar CRA and CRV. These elevations are similar to events observed in tumors elsewhere in the body 22. Retrobulbar blood flow has been well studied in glaucoma and retinal disease. In open-angle glaucoma, there is a significant lowering of the PSV and EDV in the CRA with an increase in RIa and these alterations are reversible by lowering the intraocular pressure 23. As complications of retinoblastoma, glaucoma and buphthalmos did not lower tuCRAv in the current study. The presence of the tumor itself appears to promote the increases in blood velocities that are then unaffected by the superimposed pathology.

RIa was not correlated with any specific patient or tumor characteristic. A significantly lower pulse index in the CRV was found in males and in patients with ONi. PIv is considered to be the most sensitive parameter for differentiating abnormal waveforms because it accounts for the mean velocity and because its denominator never reaches zero 11. Could tumor cell infiltration have the same effect on flow, modifying PIv, as described, in association with senile alterations of the arterial wall 15? This question should be answerable if a cut-off value is established to identify ONi. Because the difference in PIv between eyes with ONi and those without ONi was highly significant (*p*=0.009) in the present study, the number of cases needed to identify a cut-off value should not be large. Unfortunately, we had only six cases without Oni; therefore, we must postpone this investigation until future studies. Fortunately, as stated before, primary enucleation is seldom indicated; therefore, obtaining a sufficient number in both groups will take time. Another unexplained finding is the lower pulse index that was observed for males.

In conclusion, the peak blood velocities in the CRA and CRV are higher in tumor-containing eyes than in normal eyes (*p*<0.001). The arithmetic differences between the velocities in the tumor-containing and normal eyes are significant for both the CRA and the CRV (*p*=0.024), indicating that alterations are significantly higher in the CRA than in the CRV. TUvol, based on three US measurements, is related to a higher peak velocity in the CRA (*p*=0.0331). The resistivity index of the CRA is not related to the high-risk features of tumors, but the pulse index of the CRV is related to male sex (*p*=0.017) and ONi by the tumor (*p*=0.008).

## MEETING PRESENTATION

Presented at the American Academy of Ophthalmology Annual Meeting, October 2014, Chicago.

## ACKNOWLEDGMENTS

We thank Professor Giovanni G. Cerri, Chair of the Department of Radiology and Ultrasound, University of São Paulo, for allowing the use of highly specialized equipment. We also thank Mrs. Creusa M.R. Dal Bó for statistical analysis.

## Figures and Tables

**Table 1:** Patient characteristics concerning demographics, clinical and pathological classifications, tumor measurements and Doppler findings.

Patient	Age/sex	Eye/stage	pTNM	Pathm	MRIm	USm	TUvol	ONi	mCHi	tuCRAv	tuCRVv	nlCRAv	nlCRVv	RIa	PIv
1	28/M	OS/E	pT3a	18	17	20.4	2.914	N	Y	25.1	18.5	10	8.5	1	1.17
2	19/F	OS/E	pT1	17	20	19	2.25	N	N	23	22.5	N/A	N/A	0.76	1.18
3	8/M	OD/E	pT3a	20	19.7	17.3	N/A	N	Y	N/A	N/A	N/A	N/A	N/A	N/A
4	31/M	OS/D	pT2b	24	15.6	16.1	1.12	Y	N	38.5	36	N/A	N/A	0.84	0.45
5	23/M	OS/E**	pT3a§	16	22.2	20	2.99	N	Y	22.1	9.5	10.6	7.5	0.8	0.94
6	37/F	OS/D	pT1	12	12	13	0.44	N	N	23.3	10	15.3	N/A	0.78	1.27
7	12/F	OD/E*	pT2a	18	21	12.8	0.49	N	N	13	8.2	4.8	4	1	0.9
8	36/F	OS/E	pT3a	28	28	26	1.65	Y	Y	13.1	10.6	6.5	3.2	0.78	1.2
9	21/M	OD/E	pT3a	20	25	25.7	1.1	Y	N	18.9	11.2	7.6	3.5	0.83	0.53
10	48/M	OD/D	pT3a	15	21	30	2.66	Y	N	45	10	8.5	5.5	1	0.85
11	66/M	OD/D	pT3a	20	17	16.2	1.872	N	Y	29	11.4	N/A	N/A	0.88	1.14
12	48/M	OS/D	pT2a	16	14	20	2.2	Y	N	23	26	7	6.1	1	0.9
13	16/M	OS/D	pT2b	13	10.6	13.5	0.32	Y	N	30	14	8.2	7.1	0.87	0.9
14	30/M	OS/D	pT2b	20	15.9	18	2.09	Y	N	46	28.5	36	10	0.89	0.8
15	18/M	OS/D	pT2b	16	16	16.8	1.52	Y	N	13.4	8.5	4.7	3.2	0.6	0.67
16	36/M	OD/E**	pT3a	10	24.4	23.8	5.56	Y	Y	44.5	14.4	15	6.9	0.94	0.93
17	20/M	OD/E	pT3b	20	21	23.8	4.5	Y	Y	39.7	22	11	7.8	0.94	0.69
18	72/M	OD/D	pT2b	20	12.3	14.1	0.94	Y	N	19.4	10.9	13.6	10.9	0.7	0.38

Age in months; sex: M=male, F=female; stages ‘D’ and ‘E’ from the international classification (12); pTNM=AJCC pathological classification (16); Pathm, USm, MRIm=tumor size in millimeters, as measured from a pathological section, by ultrasonography and by magnetic resonance imaging, respectively; TUvol=tumor volume in cm^3^; ONi=optic nerve invasion; mCHi=massive choroid invasion; Y=presence; N=absence; tuCRAv and nlCRAv=peak blood velocities in the central retinal artery in tumorous and normal eyes, respectively; tuCRVv and nlCRVv=peak blood velocities in the central retinal vein in tumorous and normal eyes, respectively (velocity in cm/sec); RIa=resistivity index in the central retinal artery; PIv=pulse index in the central retinal vein; *=phthisis; **=proptosis; §=initially presenting with only calcification.

**Table 2:** Blood velocities in the central retinal artery and centralretinal vein in tumorous (tuCRAv and tuCRVv) and normal (nlCRAv and nlCRVv) eyes.

Variable	n	Mean	MD	Median	Min	Max	p*
tuCRAv	14	26.89	12.14	23.15	13.00	46.00	<0.001
nlCRAv	14	11.34	7.87	9.25	4.70	36.00	
tuCRVv	13	14.79	6.83	11.20	8.20	28.50	<0.001
nlCRVv	13	6.48	2.53	6.90	320	10.90	
≠CRAv	13	16.13	9.93	11.50	5.80	36.50	0.024
≠CRVv	13	8.32	5.98	7.40	0.00	19.90	

The blood velocity was higher in tumorous eyes than in normal eyes (highly significant difference). When considering the arithmetic differences between the tumorous and the normal eyes for both the artery (≠CRAv) and the vein (≠CRVv), the *P*-value was also significant, indicating that tuCRAv is more influenced by the tumor than is tuCRVv. (*) Student’s t-test for matched pairs.

**Table 3:** Correlations of the tumor size, as measured by ultrasound (USm), MRI (MRIm), and pathological sectioning (Pathm), and the tumor volume (TUvol) with the peak blood velocities in the central retinal artery (tuCRAv) and central retinal vein (tuCRVv).

		Age	Pathm	MRIm	USm	TUvol
	**n**	**17**	**16**	**17**	**17**	**17**
tuCRAv	r (*)	0.125	−0.22	−0.05	0.302	0.535
		*p*	0.633	0.406	0.844	0.24	0.027
		**n**	**13**	**13**	**13**	**13**	**13**
tuCRVv	r (*)	0.087	0.02	−0.33	0.005	0.306
		*p*	0.777	0.948	0.275	0.987	0.31

A positive correlation was found between TUvol and tuCRAv. The larger the volume of the tumor was, the faster the blood flow in the central retinal artery was. (*) Pearson’s correlation coefficient, n=number of samples studied.

**Table 4:** Correlation of optic nerve invasion by the tumor with the resistivity index of the central retinal artery and the pulse index of the central retinal vein.

		ONi		PreONi		PosONi	
		N	Y	N	Y	N	Y
	**n**	**6**	**11**	**6**	**11**	**13**	**4**
PIv	Mean/std	1.10/0.15	0.75/0.24	1.10/0.15	0.75/0.24	0.92/0.28	0.75/0.18
	Min/max	0.90/1.27	0.38/1.20	0.90/1.27	0.38/1.20	0.38/1.27	0.53/0.93
	Median	1.16	0.80	1.16	0.80	0.90	0.77
	P25/P75	0.94/1.18	0.53/0.90	0.94/1.18	0.53/0.90	0.80/1.17	0.61/0.89
	p*	0.009		0.009		0.256	
	n	6	11	6	11	13	4
RIa	Mean/std	0.87/0.11	0.85/0.12	0.87/0.11	0.85/0.12	0.84/0.12	0.93/0.07
	Min/max	0.76/1.00	0.60/1.00	0.76/1.00	0.60/1.00	0.60/1.00	0.83/1.00
	Median	0.84	0.87	0.84	0.87	0.84	0.94
	P25/P75	0.78/1.00	0.78/0.94	0.78/1.00	0.78/0.94	0.78/0.89	0.89/0.97
	p*	0.960		0.960		0.190	

Optic nerve invasion was correlated with a smaller PIv; this difference persisted when considering PreONi but did not when considering PosONi. PIv=pulsatility index of the central retinal vein; RIa=resistivity index of the central retinal artery; N=without invasion; Y=with invasion; ONi=optic nerve invasion; PreONi=prelaminar optic nerve invasion; PosONi=postlaminar optic nerve invasion. (*) non-parametric Mann-Whitney U test.
