# ERRATA

**DOI:** 10.6061/clinics/2017(08)10

**Published:** 2017-08

## CLINICS 2015;70(12):797-803

In the article **Blood flow velocity in monocular retinoblastoma assessed by color doppler**, on page 801, remove the following sentences of the **RESULTS** section:

**“The difference persisted when considering PreONi (*p*=0.0088) but not PosONi (*p*=0.2563). ONi did not modify the arterial index Rla (*p*=0.9596).”**

**and**

Replace **Table 4** and its legend for:

**Table 4:** Correlation of optic nerve invasion by the tumor with the resistivity index of the central retinal artery and the pulse index of the central retinal vein.

		ONi		PreONi		PosONi	
		N	Y	N	Y	N	Y
	**n**	**6**	**11**	**6**	**11**	**6**	**4**
PIv	Mean/std	1.10/0.15	0.75/0.24	1.10/0.15	0.75/0.24	1.10/0.15	0.75/0.18
	Min/max	0.90/1.27	0.38/1.20	0.90/1.27	0.38/1.20	0.90/1.27	0.53/0.93
	Median	1.16	0.80	1.16	0.80	1.16	0.77
	P25/P75	0.94/1.18	0.53/0.90	0.94/1.18	0.53/0.90	0.93/1.21	0.57/0.91
	p*	0.009		0.009		0.019	
	**n**	**6**	**11**	**6**	**11**	**6**	**4**
RIa	Mean/std	0.87/0.11	0.85/0.12	0.87/0.11	0.85/0.12	0.87/0.11	0.93/0.07
	Min/max	0.76/1.00	0.60/1.00	0.76/1.00	0.60/1.00	0.76/1.00	0.83/1.00
	Median	0.84	0.87	0.84	0.87	0.84	0.94
	P25/P75	0.78/1.00	0.78/0.94	0.78/1.00	0.78/0.94	0.78/1.00	0.89/0.99
	p*	0.960		0.960		0.476	

Optic nerve invasion was correlated with a smaller PIv; this difference persisted when considering PreONi but did not when considering PosONi. PIv=pulsatility index of the central retinal vein; RIa=resistivity index of the central retinal artery; N=without invasion; Y=with invasion; ONi=optic nerve invasion; PreONi=prelaminar optic nerve invasion; PosONi=postlaminar optic nerve invasion.

(*) non-parametric Mann-Whitney U test.

